# Safety of locating the tip of a medium-long catheter at the axillary front and clavicle midline: A protocol for systematic review and meta-analysis

**DOI:** 10.1097/MD.0000000000023726

**Published:** 2020-12-11

**Authors:** Yali Zhao, Jie Geng, Xing Wu, Suiting Xiong, Liwei Wang, Juanxia Wang, Haijv Ma, Fengxian Wei, Zhihong Wei

**Affiliations:** a Lanzhou University Second Hospital; b The School of Nursing, Lanzhou University, Lanzhou, Gansu, People's Republic of China.

**Keywords:** medium-long catheters, meta-analysis, safety

## Abstract

**Background:**

Medium-long catheters are being used more and more widely in clinical practice, but we still do not know the impact of different placements, but this is an important clinical issue that cannot be ignored.

**Objective:**

At present, the tip positioning of the mid-length catheter mainly includes the anterior part of the axilla and the midclavicular line. Different positioning may have different effects. Therefore, we did this research to confirm which positioning is more safety.

**Methods:**

We systematically searched the Chinese and English databases: PubMed, Embase, CENTRAL, CINAHL, Web of Science, China Knowledge Network, China Biomedical Literature Database, VIP, Wan Fang. Literature screening, data extraction, and quality evaluation were carried out by 2 researchers, and finally, use Stata to carry out meta-analysis.

**Results:**

This study is ongoing and the results will be submitted to a peer-reviewed journal for publication.

**Ethics and dissemination:**

Ethical approval is not applicable, since this is an overview based on published articles.

**Protocol registration number:**

INPLASY2020110042

## 1 Introduction

Infusion of intravenous pulses is an important way of clinical medicine, and it is also an essential way of rescue and treatment.^[1]^ At the same time, it is also the most frequently performed operation in nursing work, but repeated puncture will cause great inconvenience and pain to patients undergoing long-term infusion therapy, and various complications will also increase the workload of nursing staff and the difficulty of subsequent treatment.^[2,3]^ Therefore, it is very necessary to choose a suitable infusion method for the treatment of patients, especially for patients with long-term infusion therapy.^[4]^ Medium-length catheter is abbreviated as medium-length catheter, also called midline catheter. It is a blood access device for infusion through a peripheral vein. It is usually inserted from the expensive vein, cephalic vein, or median vein.^[5,6]^ The tip of the catheter is at the level of the armpit or under the shoulder. Suitable for medium and long-term infusion and patients with poor peripheral venous access conditions. Due to the patient's requirements for the type of infusion and the amount of infusion, the intravenous indwelling needle can no longer meet the needs of peripheral intravenous infusion treatment.^[7]^ The medium and long venous catheter is another vascular access device that enters through the peripheral vein. It is more dangerous than the intravenous indwelling needle to cause phlebitis Low, lower risk of infection than central venous catheter. Medium and long catheters are easy to insert, have little irritation, low infection rate, and have a wide range of clinical applications. All low-irritant, isotonic or nearly isotonic drugs and liquids infused through peripheral venous short tubes are suitable for medium and long catheters can better meet the patient's infusion requirements.

Systematic evaluations and meta-analyses can provide a scientific basis for health decisions and can also form higher-level recommendations in guidelines. There are many uses and studies of long and medium catheters in veins, and the main complications are phlebitis, exudation, blockage, thrombosis, displacement, and so on.^[8–12]^ Therefore, we conducted this meta-analysis to analyze the impact of different locations on security.

## 2 Methods and analysis

### 2.1 Study registration

This NMA has been registered on the International Platform of Registered Systematic Review and Meta-analysis Protocols (INPLASY). The registration number is INPLASY2020110042, DOI number is 10.37766/inplasy2020.11.0042 (https://inplasy.com/inplasy-2020-11-0042/).

### 2.2 Study inclusion and exclusion criteria

#### 2.2.1 Types of studies

The randomized controlled trial (RCT) or observational studies (cross-sectional studies, cohort studies, and case-control studies) published at home and abroad, regardless of whether blinding and allocation concealment are used, are limited to Chinese and English.

Exclusion criteria: Repeated publications; Conference abstracts; Unable to obtain full text documents; Unable to obtain relevant valid data.

#### 2.2.2 Types of participants

All patients with medium and long catheters have no restrictions on age, type of disease, or gender.

#### 2.2.3 Types of interventions

The tip of the mid-length catheter is positioned in front of the armpit, the tip of the mid-length catheter is positioned at the mid-clavicular line.

#### 2.2.4 Types of outcomes measures

Main outcomes: we mainly focus on the safety (complication) of positioning the tip of the mid-length catheter in the front of the axilla and the mid-clavicular line.

### 2.3 Search strategy

#### 2.3.1 Electronic searches

We will search the following electronic bibliographic databases: PubMed, Embase, Cochrane Library, CINAHL Complete, as well as the Chinese databases: China Knowledge Network, China Biomedical Literature Database, VIP Data, Wan Fang Data. The time is from the construction of the database to December 2019. English search term is “medium-long catheter.”

#### 2.3.2 Other resources

**Figure 1:**
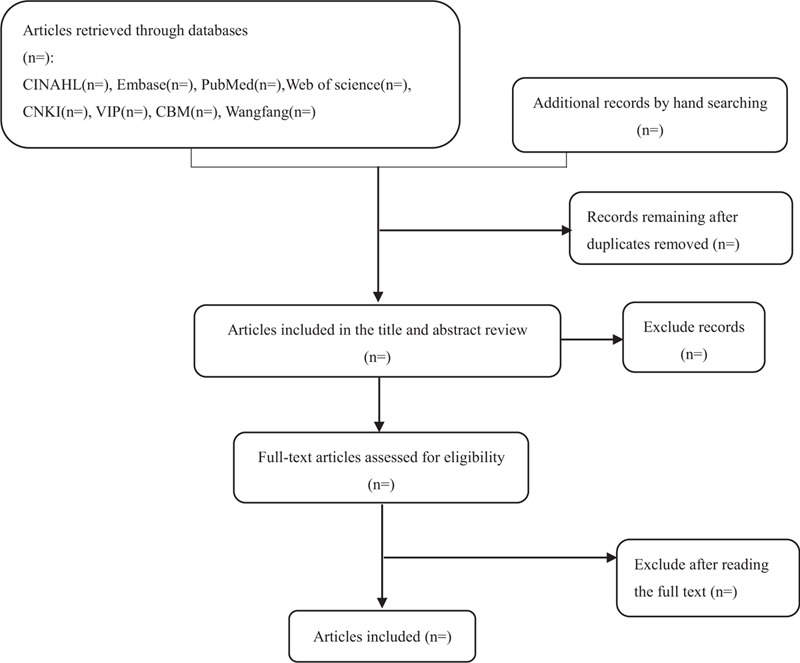
Summary of evidence search and selection.

Searches of the grey literature, and the bibliographies of relevant papers were also used to complement the results of the database searches. The detailed search flowchart is shown in Figure 1.

#### 2.3.3 Search strategies

Take PubMed as an example, the search strategy is shown in Table 1

**Table 1:** Searching strategy in PubMed.

#1	randomized controlled trial [pt]
#2	controlled clinical trial [pt]
#3	randomized [tiab]
#4	placebo [tiab]
#5	clinical trials as topic [mesh: noexp]
#6	randomly [tiab]
#7	trial [ti]
#8	#1 OR #2 OR #3 OR #4 OR #5 OR #6 OR #7
#9	animals [mh] NOT humans [mh]
#10	#8 NOT #9
#11	“observational” [tiab]
#12	observational studies as topic [mh]
#13	observational study [pt]
#14	“prospective” [tiab]
#15	prospective study [mh]
#16	“retrospective” [tiab]
#17	retrospective study [mh]
#18	“cohort” [tiab]
#19	cohort studies [mh]
#20	“cross-sectional” [tiab]
#21	cross sectional study [mh]
#22	“case control” [tiab]
#23	case control study [mh]
#24	“case series” [tiab]
#25	“epidemiologic” [tiab]
#26	“epidemiological” [tiab]
#27	epidemiologic studies [mh]
#28	#11 OR #12 OR #13 OR #14 OR #15 OR #16 OR #17 OR #18 OR #19 OR #20 OR #21 OR #22 OR #23 OR #24 OR #25 OR #26 OR #27
#29	medium-long catheter [tiab]
#30	(#10 OR #28) AND #29

#### 2.3.4 Literature screening

All search results are imported into EndNote X8 literature management software, 2 reviewers (JG, YLZ) will screen the titles and abstracts of literature independently, then read the full text to assess literature according to the inclusion and exclusion criteria, any disagreements will be resolved by a third reviewer (ZHW).

#### 2.3.5 Data extraction

Two researchers independently screened the literature and extracted data according to the established inclusion and exclusion criteria, and solicited the opinions of a third party to resolve in case of differences. Independently, 2 review authors will extract the following data: literature title, publication year, research design, subjects, demographic characteristics, sample size and outcome indicators, and so on.

### 2.4 Study quality assessment

Two researchers evaluated the quality of the included RCT based on the risk of bias evaluation tool recommended by the Cochrane Collaboration.^[15]^ The evaluation content mainly includes the following 6 aspects: random allocation method; allocation plan concealment; blind method; completeness of the result data; selective reporting of research results; other sources of bias. According to the results of each study, the above 6 items need to be judged as “yes (low bias),” “no (high bias),” “unclear (lack of relevant information or uncertain risk of bias).” The cross-sectional research literature quality evaluation adopts the evaluation standards recommended by the Agency for Healthcare Research and Quality, and contains a total of 11 items, which are evaluated as “yes,” “no,” and “unclear” respectively. Use the Newcastle–Ottawa scale to evaluate the quality of the included cohorts and case-control studies.^[16]^

### 2.5 Statistical analysis

#### 2.5.1 Data synthesis

The data were meta-analyzed by Stata software, count data uses odds ratio as the effect indicator, and measurement data uses mean difference as the effect indicator. Each effect size is given its point estimate and its 95% confidence interval(confidence intervals, CI). For the results of the number of studies more than 10 items, Stata12.0 software was used to draw a funnel chart and combined with Egger test to publish the bias. *P* < .05 indicates that the difference is statistically significant.

#### 2.5.2 Assessment of heterogeneity

The Cochrane *Q* test and *I*^2^ were used to conduct qualitative and quantitative analysis of statistical heterogeneity, and the test level *α* = 0.1, that is, if *P* < .1 or *I*^2^ > 50%, it indicates that there is heterogeneity among the included studies, and the random effects model is used; If *P* > .1 and *I*^2^ < 50%, it indicates that the possibility of heterogeneity between studies is small, and fixed-effects model meta-analysis is used.

#### 2.5.3 Subgroup analysis

If the evidence is sufficient, we will conduct a subgroup analysis to determine the difference between different article type, different age, different gender, and so on.

### 2.6 Quality of evidence

Finally, we evaluate each result according use the GRADE (Grading of Recommendations Assessment, Development, and Evaluation). The evidence levels classified into 4 levels: high, moderate, low, or very low.

## 3 Discussion

For patients with poor peripheral venous conditions and expected long infusion time, early use of medium-length catheters can reduce repeated punctures, relieve patient pain, reduce nurses’ workload, and increase economic benefits. It is worthy of clinical promotion if it meets clinical indications. However, there is currently no research on the position of the medium and long catheters, so we do this research to provide clinical evidence.

### 3.1 Uncited references

^[13,14]^.

## Author contributions

**Conceptualization:** Yali Zhao, Zhihong Wei.

**Data curation:** Zhihong Wei.

**Formal analysis:** Suiting Xiong.

**Funding acquisition:** Xing Wu.

**Investigation:** Liwei Wang.

**Methodology:** Jie Geng, Liwei Wang.

**Project administration:** Juanxia Wang.

**Resources:** Juanxia Wang.

**Software:** Haijv Ma.

**Supervision:** Haijv Ma.

**Validation:** Fengxian Wei.

**Visualization:** Fengxian Wei.
